# Genetic and Functional Dissection of ARMS2 in Age-Related Macular Degeneration and Polypoidal Choroidal Vasculopathy

**DOI:** 10.1371/journal.pone.0053665

**Published:** 2013-01-09

**Authors:** Yong Cheng, LvZhen Huang, Xiaoxin Li, Peng Zhou, Wotan Zeng, ChunFang Zhang

**Affiliations:** 1 Department of Ophthalmology, People’s Hospital, Peking University, Beijing, China; 2 Key Laboratory of Vision Loss and Restoration, Ministry of Education, Beijing, China; 3 Department of Ophthalmology, Eye and ENT Hospital of Fudan University, Shanghai, China; 4 Chinese National Human Genome Center, Beijing, China; 5 Department of Clinical Epidemiology, People’s Hospital, Peking University, Beijing, China; National Central University, Taiwan

## Abstract

Age-related maculopathy susceptibility 2(*ARMS2*) was suggested to be associated with neovascular age-related macular degeneration (nAMD) and polypoidal choroidal vasculopathy (PCV) in multiple genetic studies in Caucasians and Japanese. To date, no biological properties have been attributed to the putative protein in nAMD and PCV. The complete genes of *ARMS2* and *HTRA1* including all exons and the promoter region were assessed using direct sequencing technology in 284 unrelated mainland northern Chinese individuals: 96 nAMD patients, 92 PCV patients and 96 controls. Significant associations with both nAMD and PCV were observed in 2 polymorphisms of *ARMS2* and *HTRA1* rs11200638, with different genotypic distributions between nAMD and PCV (*p*<0.001). After adjusting for rs11200638, *ARMS2* rs10490924 remained significantly associated with nAMD and PCV (*p*<0.001). Then we overexpressed wild-type ARMS2 and ARMS2 A69S mutation (rs10490924) in RF/6A cells and RPE cells as in vitro study model. Cell proliferation, attachment, migration and tube formation were analyzed for the first time. Compare with wild-type ARMS2, A69S mutation resulted in a significant increase in proliferation and attachment but inhibited cell migration. Moreover, neither wild-type ARMS2 nor A69S mutation affected tube formation of RF/6A cells. There is a strong and consistent association of the *ARMS2/HTRA1* locus with both nAMD and PCV, suggesting the two disorders share, at least partially, similar molecular mechanisms. Neither wild-type ARMS2 nor A69S mutation had direct association with neovascularisation in the pathogenesis of AMD.

## Introduction

Age-related macular degeneration (AMD) causes irreversible central vision loss and is the leading cause of blindness in the elderly population, characterized as chronic and progressive degeneration of photoreceptors, the underlying retinal pigment epithelium (RPE), Bruch’s membrane, and possibly, the choriocapillaris in the macula. [1], [2], [3], [4], [5] AMD is divided clinically into dry and wet AMD. The “wet” form of the disease or neovascular, characterized by the development of choroidal neovascular (CNV) membranes, is the main cause of visual impairment in macular degeneration. [6].

Polypoidal choroidal vasculopathy (PCV) is a macular disease found in the elderly that is as prevalent as exudative AMD in the Asian population, accounting for approximately 30% to 50% of the total number of eyes with senile macular diseases in elderly Asians [7], [8]. It is characterized by an abnormal choroidal vascular network with characteristic aneurismal dilations at the border of the vascular network [9], [10]. The incidence of PCV in the Chinese and Japanese populations with neovascular AMD has been reported to be 24.5% and 54.7% respectively, compared with a much lower incidence in Caucasians [10], [11], [12]. PCV has been described as a separate clinical entity differing from AMD and other disease associated with subretinal neovascularization and it remains controversial as to whether or not PCV represents a sub-type of nAMD [10].

Initial efforts to investigate the genetic basis of AMD utilized family studies. A concordance for AMD phenotypes in twins, and a higher risk of siblings of individuals with AMD have been reported [13], [14], [15], [16], [17], [18]. These early studies lead to genome-wide linkage analyses using microsatellite markers to search for chromosomal regions associated with affected individuals [19], [20], [21], [22], [23], [24], [25], [26], [27]. Several candidate regions including 1q32 and 10q26 were confirmed by a metaanalysis [28]. Progress in genotyping and sequencing technology extended detailed genetic association studies to the entire genome. Age-related eye disease studies (AREDS) of AMD case-control subjects using 100,000 SNPs resulted in the identification of four chromosomal regions significantly associated with the disease, namely complement factor H (CFH) (1q32), the age-related maculopathy susceptibility 2(*ARMS2*)/Htra serine peptidase 1 (*HTRA1*) (10q26), complement component 2/complement factor B (C2/BF, 6p21), and complement component 3 (C3, 19p13). Based on the reported AMD-associated genes, genetic studies have been initiated to investigate the molecular mechanisms underlying nAMD and PCV. Recently, numerous studies by direct examinations of single nucleotide polymorphism (SNP) in chromosomal regions identified by genome-wide linkage analysis have presented several genes have been reported to be strongly associated with these two diseases, including complement factor H [29], [30], [31], [32], *ARMS2* and *HTRA1* genes [33], [34], [35], [36], [37], [38]. The association between AMD,PCV and three SNPs in these gene regions, namely rs1061170 (CFH), rs10490924 (ARMS2), and rs11200638 (HTRA1), were verified by a number of research groups in Caucasians and Japanese [33], [36], [37], [38], [39], [40], [41], [42], [43].

There is strong linkage disequilibrium (LD) across the ARMS2-HTRA1 region, making genetic association studies alone insufficient to distinguish between the two candidates. Instead, a comprehensive characterization of AMD-associated variants in the region of high LD is warranted, closely accompanied by a sophisticated analysis of their possible functional relevance in the disease process. Nevertheless, reports of a causal variant in the promoter region of HTRA1 [33], [34] could not be verified by others [37]. In contrast, ARMS2 is an evolutionarily recent gene within the primate lineage and, so far, no biological properties have been attributed to the putative protein.

In this study, we investigated the genetic determinants of nAMD and PCV to highlight their genetic differentiation. We sequenced the entire *ARMS2* and *HTRA1* gene including all exons and the promoter region. Our intention was to investigate whether these associations occur in Chinese patients with nAMD and PCV from Northern Chinese and second, sought to investigate the biological function of *ARMS2*.

## Materials and Methods

### Subjects

Two hundred and eighty-four unrelated northern Chinese were studied (Table 1); 96 patients had neovascular Age-Related Macular Degeneration (nAMD) (mean age ± standard deviation [SD], 70.3±8.8 years; ratio of men to woman, 64.6∶35.4×) and 92 patients had Polypoidal Choroidal Vasculopathy (PCV) (mean age ± SD, 69.5±9.4 years; ratio of men to woman, 52.2∶47.8). For controls, 96 individuals without age-related maculopathy (ARM) were studied (mean age ± SD, 67±9.5 years; ratio of men to woman, 44.8∶55.2). They were recruited at the Department of Ophthalmology in the Peking University People’s Hospital. The study was approved by the Ethnic Committee of Peking University People’s Hospital. An informed consent process was established following the guidelines of the Helsinki Declaration, and consent forms were signed by all subjects. All subjects received a standard ophthalmic examination, including visual acuity measurement, slit-lamp biomicroscopy, and dilated fundus examination that performed by a retinal specialist. All cases with Age-Related Macular Degeneration (AMD) and Polypoidal Choroidal Vasculopathy (PCV) underwent fluorescein angiography, optic coherence tomography (OCT), and indocyanine green angiograms with HRA2 (Heidelberg Engineering, Heidelberg, Germany). Diagnosis of neovascular AMD (nAMD) or age related maculopathy (ARM) was defined by International Classification System for ARM. [44] The diagnosis of PCV was based on indocyanine green angiography (ICGA) results, which showed a branching vascular network that terminated in aneurysmal enlargements, that is, polypoidal lesions. Eyes with other macular abnormalities, such as pathologic myopia, idiopathic choroidal neovascularization (CNV), presumed ocular histoplasmosis, angioid streaks, and other secondary CNV, were excluded. Normal controls were defined as no clinical evidence of early or late AMD in either eye or any other eye diseases except mild age-related cataract. Subjects with severe cataracts were excluded from the study.

**Table 1:** Characteristics of the study population.

	nAMD	PCV	Controls	P
Total	96	92	96	
Males.n(%)	62(64.6)	48(52.2)	43(44.8)	>0.05
Female.n(%)	34(35.4)	44(47.8)	53(55.2)	
Mean age (±SD)(yrs)	70.3±8.8	69.5±9.4	67±9.5	0.08

### Genomic DNA Extraction and PCR Amplification

Genomic DNAs were extracted from venous blood leukocytes with a genomic extraction kit (Beijing eBios Biotechnology Co., Ltd). A PCR amplification kit was purchased from Dingguo Biotechnology (Beijing) Co., Ltd. The PCR primers were designed by using Primer 3 online design tool and synthesized by Invitrogen Corporation Shanghai. A PCR amplification was performed in 50 µL reactions containing 50 ng genomic DNA, 20µM primers, 32 µL ddH_2_O, 2.5 mM dNTPs, 2.0U Taq, and 5 µL 10×PCR Buffer. Thermo-cycling was carried out with an initial denaturation step of 94C for 5 min, followed by 10 cycles of 94 C for 30 s, 60 C for 1 min and 72C for 45 s; then 30 cycles of 94C for 30 sec, 55C for 1 min, and 72C for 45 s; and ended with a final single extension at 72C for 3 min. The PCR products were assessed by 1.0% agarose gel electrophoresis; then DNA bands with correct sizes were purified with a 96-well PCR purification kit (MILLIPORE, USA).

### Genotyping by Sequencing

All above purified PCR products were directly sequenced with ABI 3730XL DNA sequencer. Variants in *ARMS2* and HTRA1 genes were identified by an ABI automatic allele calling software. Genotyping had 99% completeness and 99% accuracy as determined by random re-sequencing of 10% samples.

### Plasmid Constructs

The entire open reading frame of the wild-type human ARMS2 gene (XM_001131263) was amplified from ARPE19 cells by RT-PCR using gene-specific primers (forward: CACACTCCATGATCCCAGCTTCTAAAATCCACACTGAGCTCTGC-3; reverse: GCAGAGCTCAGTGTGGATTTTAGAAGCTGGGATCATGGAGTGTG-3) and was subsequently cloned in pcDNA3.1-CT-GFP to construct a human wild-type ARMS2 expression vector using the pcDNATM3.1 Directional TOPO expression kit (Invitrogen, Carlsbad, CA, USA) according to the manufacturer’s instructions. All constructs were verified before use by direct sequencing. Plasmid containing the ARMS2 A69S mutation (rs10490924, G270T in mRNA) was made by site-directed mutagenesis using oligonucleotides annealed to the target sequence and the QuikChange kit (Stratagene Santa Clara, CA, USA).

### Cell Culture and Reagents

RF/6A cells (CRL-1780 cell line) and human RPE cells (ARPE-19 cell line) were obtained from the American Tissue Culture Collection (Manassas, VA) and were cultured in Dulbecco’s Modified Eagle Media (DMEM) with 10% fetal bovine serum (FBS, Gibco, Invitrogen, Grand island, NY), 100 units/ml penicillin, 100 µg/ml streptomycin (Sigma, St. Louis, MO) at 37°C under 5% CO_2_, and 95% humidified air. Before hypoxia, the media was replaced with DMEM free of serum. The cells were then incubated overnight and perfused with 1% O_2_, 94% N^2^, and 5% CO_2_ in a CO_2_ incubator for 24 h [45]. Cells were transfected with plasmid DNA (pcDNA3.1-CT-GFP–270G/T as test vectors and pReceiver-M29-Basic as a negative control). Transfections were performed using Lipofectamine 2000 reagent (Invitrogen) according to the manufacturer’s protocol.

### RT-PCR and Relative Quantitative Real-time PCR

Total RNA was isolated using a TRIzol reagent (Invitrogen). The first strand of cDNA was synthesized with 1 ug of total RNA, oligo(dT)_15_ primer, and AMV reverse transcriptase (Promega). The primers used in RT-PCR were ARMS2: forward, 5′- GGTCTAGATAGTTATTAATAGTAATCAAT -3′; reverse, 5′- GAATTCACCTTGCTGCAGTGTGGATG -3′; and β-actin: forward, 5′-AGCGGGAAATCGTGCGTG-3′; reverse, 5′-CAGGGTACATGGTGGTGCC-3′ Real-time PCR reactions were performed with SYBR Green PCR master mix (Roche, Basel, Switzerland). The specificity of the PCR amplification products was checked by performing dissociation melting-curve analysis and by 1% agarose gel electrophoresis. Quantification analysis of BMP4 mRNA was normalized with a housekeeping gene, β-actin, as an internal control. Relative multiples of changes in mRNA expression were determined by calculating 2^−ΔΔct.^

### Western Blot Analysis

Proteins were extracted from cultured RF/6A cells and ARPE-19 cells using T-PER tissue protein extraction reagent (Pierce, Rockford, IL, USA), and the protein concentration was measured using the Bio-Rad protein assay kit (Bio-Rad, Hercules, CA, USA). Equal amounts of protein lysate (20–60 ug) were resolved on 10% Tris-HCl polyacrylamide gels and then transferred to a PVDF blotting membrane (Millipore, Billerica, MA, USA). After blocking, each membrane was incubated with antibodies specific for human ARMS2 and β-actin (Abcam, Cambridge, MA, USA). After incubation with peroxidase-conjugated goat anti-rabbit secondary antibodies (ZSGB-Bio, Beijing, China), protein bands were detected by chemiluminescence (Pierce). Western blots were repeated three times and qualitatively similar results were obtained.

### Cell Proliferation Assay

To assess cell proliferation, a 3-[4,5-dimethylthiazol-2-yl]-2,5-diphenyltetrazolium bromide (MTT; Roche, Molecular Biochemicals, Mannheim, Germany) assay was used. Briefly, RF/6A cells and ARPE-19 cells were plated at a density of 2×10^3^ cells per well in 96-well culture plates. After attachment, the culture medium was changed to DMEM containing 10% FBS, and the cells were incubated for 24 h. After reaching 80% confluence, the cells were starved with DMEM containing 1% FBS for 6 h and then were transfected with a mixture of Lipofectamine 2000 (Invitrogen) and plasmids pcDNA3.1-CT-GFP–270G/T, pReceiver-M29-Basic, respectively. After 24 h, MTT was added to the culture medium and the cells were incubated for an additional 4 h. Formazan crystals that formed were then dissolved by the addition of dimethyl sulfoxide (100 µl/well). Absorbance at 570 nm was measured using an ELISA plate reader (Dynatech Medica, Guernsey, UK) [46]. Cell proliferation was measured by a modified MTT assay on 24, 48, 72, and 96 h. Media were changed on day 3. Each experiment was undertaken using three wells and was performed at least three times.

### Cell Attachment Assay

Ninety-six-well plates coated with 1.25 µg/ml fibronectin in 100 µl of PBS were put into the incubator overnight at 4°C. Transfected cells (1×104) were trypsinized, added to each well, and allowed to attach for 6 h [47]. The cells were then washed gently twice with PBS, and 150 µl fresh medium was added to each well with MTT. The absorbance was measured with an ELISA plate reader at 570 nm. We used three different wells to detect the cell attachment and repeated all the experiments three times.

### Cell Migration

Migration assay was performed as described before [48]. Briefly, 2×104 cells were placed in the upper chamber in a final volume of 200 µl of serum-free medium. Next 10% FBS was placed in the bottom chamber for a final volume of 600 µl. All migration assays were conducted for 6 h at 37°C. At the end of the assay, the cells were fixed in 4% PFA and stained with DAPI for 15 min. Remaining cells were wiped away with a cotton bud, and the membrane was imaged. The number of cells from five random fields of view was counted.

### Tube Formation

The tube formation assay was conducted to investigate the effect of pcDNA3.1-CT-GFP–270G/T and pReceiver-M29-Basic on RF/6A in vitro. Aliquots (150 µl) of matrigel solution were poured into the 48 well plates (repeated 2 more times), and the plates were incubated at 37°C for 1 h in a 5% CO2 incubator to form a matrigel gel [49]. RF/6A cells (1×104 per well) treated with siRNA for 48 h were seeded on the matrigel and cultured in DMEM medium. The networks in matrigel from five randomly chosen fields were counted and photographed under a microscope.

### Flow Cytometry

Apoptosis was measured with a FITC Annexin V Apoptosis Detection Kit (BD Science, US, Cat# 556547), according to the manufacturer’s instructions. Briefly, ARPE-19 cells (1×10^5^) after transfection were seeded in six-well plates and incubated for 24 h, 48 h, 72 h and 96 h. Then, the cells were detached with EDTA, washed in cold phosphate buffered saline (PBS), and stained with Annexin-V-FITC and propidium iodide (P.I.), according to the manufacturers’ instructions. Flow cytometry analysis was immediately performed (ex/em = 488/530 nm). The samples were analyzed by flow cytometer (FACSCalibur; BD Biosciences, Franklin Lakes, NJ) with Cell Quest software (BD, Biosciences). Then, 10^4^ cells were collected and divided into four groups: dead cells (Annexin V−/PI+, UL), late apoptotic cells (Annexin V+/PI+, UR), viable cells (Annexin V−/PI-, LL), and early apoptotic cells (Annexin V+/PI-, LR). The apoptotic rate was calculated as the percentage of early apoptotic cells (LR) plus late apoptotic cells (UR).

ARPE-19 cells (1×10^5^) after transfection were seeded in six-well plates and incubated for 48 h. Cells were detached using ethylene diamine tetraacetic acid (EDTA), washed in ice-cold PBS (4°C), and treated with the BD Cycletest™ Plus DNA Reagent Kit (Becton Dickinson) according to the manufacturer’s protocol. Samples were analyzed using a FACS Caliber cytometer (Becton Dickinson). Three samples were used per experiment, and each experiment was repeated.

### Statistical Analysis

All the identified polymorphisms were assessed for Hardy-Weinberg equilibrium using χ^2^ analysis. Allelic and genotypic distributions among different groups were compared using the χ^2^ test or Fisher’s exact test, and logistic regression analysis was performed to identify the strongest associated SNPs in the *ARMS2/HTRA1* locus (SPSS, version 16.0; SPSS Science, Chicago, IL). LD and haplotype-based association analyses were performed (Haploview, version 4.2). All experiments were repeated ≥3 times. Statistical analyses were performed with Student’s *t* test. Values are expressed as mean±SD. Values of *P*<0.05 were considered statistically significant.

## Results

### SNP Analysis

The complete gene of *ARMS2* including all exons and the promoter region were sequenced. Four polymorphisms (three single nucleotide polymorphisms [SNPs] including two novel SNPs: c.147G>T & c.148T>A, and one insertion) in which there was statistically significant among AMD patients, PCV patients and control subjects were identified. The distributions of the polymorphisms and genotypes of the *ARMS2* gene for the 284 participants were presented in Table 2 and 3.

**Table 2:** Polymorphisms in the *ARMS2* gene lesion: Distribution and Genotypes in neovascular Age-Related Macular Degeneration (nAMD), Polypoidal Choroidal Vasculopathy, and Controls in the northern Chinese Population.

Marker	Relative	Risk	WT	Risk Allele Frequency				*p* value* & OR(95%CI)**		
	Position	Allele	Allele	nAMD	PCV	Controls		nAMD-Control	PCV-Control	nAMD-PCV
c.147G>T	Exon 1	T	G	0.43	0.31	0.39	*p* values	0.425	0.099	0.016
							OR (95%CI)	1.18(0.78–1.78)	0.69(0.45–1.07)	0.59(0.38–0.91)
c.148T>A	Exon 1	A	T	0.43	0.31	0.49	*p* values	0.211	3.17×10^−4^	0.016
							OR (95%CI)	0.77(0.52–1.19)	0.45(0.29–0.70)	0.59(0.38–0.91)
rs10490924	Exon 1	T	G	0.71	0.69	0.25	*p* values	3.49×10^−19^	7.98×10^−15^	0.309
							OR (95%CI)	7.34(4.66–11.57)	5.81(3.67–9.20)	0.79(0.50–1.24
EU 427528	Intron 1	TG	–	0.70	0.66	0.26	*p* values	1.31×10^−17^	4.22×10^−14^	0.419
(310–311)							OR (95%CI)	6.61(4.22–10.37)	5.50(3.49–8.69)	0.83(0.53–1.30)

* : *p*-Value <0.05 is considered to be statistically significant and they are shown in bold.

** : OR(95%CI): Odds ratios are given for the risk allele compared with the wildtype allele.

**Table 3:** Polymorphisms in the *ARMS2* gene lesion: Distribution and Allele in neovascular Age-Related Macular Degeneration (nAMD), Polypoidal Choroidal Vasculopathy, and Controls in the northern Chinese Population.

SNP	Genotype Distribution (%)				nAMD Vs Controls			PCV Vs Controls			nAMD Vs PCV		
		nAMD	PCV	Controls	Genotypic	Homo*	Heter**	Genotypic	Homo*	Heter**	Genotypic	Homo*	Heter**
c.147G>T	GG	13(13.8)	32(38.6)	21(21.9)	NA	NA	0.148	NA	NA	0.015	NA	NA	1.63×10^−4^
	GT	81(86.2)	51(61.4)	75(78.1)		NA	1.75		NA	0.45		NA	0.26
							(0.82–3.73)			(0.23–0.86)			(0.12–0.53)
	TT$	0	0	0									
	TT	13(13.8)	32(38.6)	1(1.0)	NA	NA	7.43×10^−4^	NA	NA	1.09×10^−10^	NA	NA	1.63×10^−4^
c.148 T>A	TA	81(86.2)	51(61.4)	95(99.0)		NA	0.07		NA	0.02		NA	3.91
							(0.01–0.51)			(0.002–0.13)			(1.88–8.14)
	AA$	0	0	0									
	GG	10(10.6)	13(15.7)	58(61.1)	9.94×10^−14^	2.06×10^−14^	6.11×10^−7^	1.97×10^−10^	3.90×10^−11^	3.06×10^−5^	0.585	0.3	0.427
rs 10490924	GT	34(36.2)	30(36.1)	26(27.4)		26.36	7.59		16.22	5.15		1.63	1.47
						(10.34–67.23)	(3.24–17.63)		(6.61–39.84)	(2.32–11.44)		(0.65–4.09)	(0.56–3.85)
	TT$	50(53.2)	40(48.2)	11(11.5)									
	NO^#^	11(11.7)	13(15.7)	57(60.0)	9.57×10^−13^	2.47×10^−12^	1.61×10^−6^	4.60×10^−10^	9.59×10^−10^	3.54×10^−5^	0.719	0.417	0.546
EU427528	HE^#^	35(37.2)	31(37.3)	27(28.4)		17.77	6.72		12.21	5.03		0.69	0.75
(310–311)						(7.38–42.75)	(2.97–15.22)		(5.18–28.80)	(2.28–11.13)		(0.28–1.70)	(0.29–1.91)
	HO###	48(51.1)	39(47.0)	11(11.6)									

After Bonferroni correction the represent significance at P<0.05/8 = 0.00625.

* Homozygous;Comparing the likelihood of individuals with two copies of the risk allele versus individuals with no copies of the risk allele;

** Hetrozygous;Comparing the likelihood of individuals with one copy of the risk allele versus individuals with no copies of the risk allele;

$ homozygous for the risk factor.

## HE: Hetrozygous insertion.

### HO: Homozygous insertion.

There was no significant difference in c.147G>T both between AMD patients and controls (*p* = 0.425; OR [95%CI] = 1.18 [0.78–1.78]) and between PCV patients and controls (*p* = 0.099; OR [95%CI] = 0.69 [0.45–1.07]). However, there was statistical significance between AMD patients and PCV patients (*p* = 0.016; OR [95%CI] = 0.59 [0.38–0.91]). The frequency of risk T allele (AMD = 0.43. PCV = 0.31 & controls = 0.39) showed there was little difference among them. Therefore, the risk T allele had low association with AMD or PCV diseases. The similar results presented in c.148T>A. Comparing with c.147G>T, there was statistically significant difference between PCV patients and controls (*p* = 3.17×10^−4^) while the OR (95%CI) was 0.45 (0.29–0.70).

The significant association was found in rs10490924 and EU427528 (310–311). For rs10490924, there was a great difference both between AMD patients and controls (*p* = 3.49×10^−19^; OR [95%CI] = 7.34[4.66–11.57]) and between PCV patients and controls (*p* = 7.98×10^−15^; OR [95%CI] = 5.81[3.67–9.20]). The frequency of risk T allele was much higher in AMD patients (0.71) and in PCV patients (0.69) than in control subjects (0.25). It suggested that the risk allele was tightly associated with AMD and PCV in northern Chinese population. Meanwhile, the almost same results were presented in EU427528 (310–311). There was also a greatly significant difference both between AMD patients and controls (*p* = 1.31×10^−17^; OR [95%CI] = 6.61[4.22–10.37]) and between PCV patients and controls (*p* = 4.22×10^−14^; OR [95%CI] = 5.50[3.49–8.69]). The frequency of TG insertion in AMD patients and in PCV patients were 0.70 and 0.66, respectively, whereas the frequency in controls was 0.26. The risk allele of TG insertion had a strongly increased risk of developing AMD and PCV. However, between AMD patients and PCV patients, there was no statistical significance both in rs10490924 (*p* = 0.309; OR [95%CI] = 0.79[0.50–1.24]) and in EU427528 (310–311) (*p* = 0.419; OR [95%CI] = 0.83[0.53–1.30]).

The complete gene of *HTRA1* were also sequenced. Four polymorphisms in which there was statistically significant among AMD patients, PCV patients and control subjects were identified. The distributions of the polymorphisms and genotypes of the *HTRA1* gene for the 284 participants were presented in Table 4 and 5.

**Table 4:** Polymorphisms in the *HTRA1* gene lesion: Distribution and Genotypes in neovascular Age-Related Macular Degeneration (nAMD), Polypoidal Choroidal Vasculopathy, and Controls in the northern Chinese Population.

Marker	Relative	Risk	WT	Risk Allele Frequency				*p* value* & OR(95%CI)**		
	Position	Allele	Allele	nAMD	PCV	Controls		nAMD-Control	PCV-Control	nAMD-PCV
rs11200638	Promoter	A	G	0.73	0.65	0.45	*P* values	1.42×10^−7^	1.05×10^−4^	0.157
							OR (95%CI)	3.13(2.03–4.82)	2.28(1.50–3.46)	0.73(0.47–1.13)
rs55928386	Promoter	T	C	0.03	0.05	0.07	*P* values	0.050	0.379	0.267
							OR (95%CI)	0.36(0.13–1.04)	0.68(0.28–1.62)	0.54(0.18–1.63)
rs2672598	Promoter	C	T	0.85	0.79	0.73	*P* values	0.004	0.177	0.121
							OR (95%CI)	2.14(1.27–3.61)	1.40(0.86–2.26)	1.53(0.89–2.64)
rs72171000	Promoter	–	TGTT	0.16	0.11	0.09	*P* values	0.064	0.655	0.162
							OR (95%CI)	1.79(0.96–3.34)	1.17(0.60–2.28)	0.65(0.36–1.19)

* *p*-Value <0.05 is considered to be statistically significant and they are shown in bold.

** OR(95%CI): Odds ratios are given for the risk allele compared with the wildtype allele.

**Table 5:** Polymorphisms in the *HTRA1* gene lesion: Distribution and Allele in neovascular Age-Related Macular Degeneration (nAMD), Polypoidal Choroidal Vasculopathy, and Controls in the northern Chinese Population.

SNP	Genotype Distribution (%)				nAMD Vs Controls			PCV Vs Controls			nAMD Vs PCV		
		nAMD	PCV	Controls	Genotypic	Homo*	Heter**	Genotypic	Homo*	Heter**	Genotypic	Homo*	Heter**
rs11200638	GG	11(11.8)	9(9.8)	30(32.3)	6.24×10^−6^	4.74×10^−6^	0.115	3.53×10^−4^	8.23×10^−5^	2.19×10^−3^	0.048	0.779	0.212
	GA	30(32.3)	46(50.0)	42(45.2)		6.75	1.95		5.87	3.65		0.87	1.87
						(2.87–15.91)	(0.85–4.49)		(2.35–14.70)	(1.55–8.58)		(0.33–2.31)	(0.69–5.06)
	AA$	52(55.9)	37(40.2)	21(22.5)									
	CC	88(94.6)	84(91.3)	79(85.9)	NA	NA	0.045	0.228	NA	0.163	0.491	NA	0.525
rs55928386	CT	5(5.4)	7(7.6)	13(14.1)		NA	0.35			0.51		NA	1.47
							(0.12–1.01)		NA	(0.19–1.33)			(0.45–4.80)
	TT$	0	1(1.1)	0									
	TT	3(3.2)	4(4.4)	4(4.4)	4.60×10^−3^	0.386	0.636	0.24	0.774	0.675	0.262	0.553	0.933
rs2672598	TC	21(22.6)	30(32.6)	41(44.6)		1.96	0.68		1.23	0.73		0.63	1.07
						(0.42–9.15)	(0.14–3.34)		(0.29–5.20)	(0.17–3.16)		(0.14–2.93)	(0.22–5.29)
	CC$	69(74.2)	58(63.0)	47(51.0)									
	NO^#^	65(68.4)	73(79.3)	80(84.2)	2.17×10^−3^	NA	0.002	0.29	0.369	0.218	0.11	NA	0.066
rs72171000	HE^##^	30(31.6)	18(19.6)	12(12.6)		NA	3.08		0.37	1.64		NA	0.53
							(1.46–6.48)		(0.04–3.59)	(0.74–3.65)			(0.27–1.05)
	HO###	0	1(1.1)	3(3.2)									

After Bonferroni correction the represent significance at P<0.05/8 = 0.00625.

* Homozygous;Comparing the likelihood of individuals with two copies of the risk allele versus individuals with no copies of the risk allele;

** Hetrozygous;Comparing the likelihood of individuals with one copy of the risk allele versus individuals with no copies of the risk allele;

$ homozygous for the risk factor.

_##_HE: Hetrozygous insertion.

### HO: Homozygous insertion.

The significant association was found in rs11200638. For rs11200638, there was a great difference both between AMD patients and controls (*p* = 1.42×10^−7^; OR [95%CI] = 3.13[2.03–4.82]) and between PCV patients and controls (*p* = 1.05×10^−4^; OR [95%CI] = 2.28[1.50–3.46]). The frequency of risk G allele was much higher in AMD patients (0.73) and in PCV patients (0.65) than in control subjects (0.45). It suggested that the risk allele was tightly associated with AMD and PCV in northern Chinese population.

There was no significant difference in rs55928386 both between PCV patients and controls (*p* = 0.379; OR [95%CI] = 0.68 [0.28–1.62]) and between AMD patients and PCV (*p* = 0.267; OR [95%CI] = 0.54 [0.18–1.63]).However, there was statistical significance between AMD patients and controls (*p* = 0.05; OR [95%CI] = 0.36 [0.13–1.04]). The frequency of risk T allele (AMD = 0.05. PCV = 0.379 & controls = 0.267) showed there was difference between AMD patients and controls and little difference between PCV patients and controls. Therefore, the risk T allele had association with AMD. The similar results presented in rs2672598.

There was no significant difference in rs72171000 both between AMD patients and controls (*p* = 0.064; OR [95%CI] = 1.79 [0.96–3.34]) and between PCV patients and controls (*p* = 0.655; OR [95%CI] = 1.17 [0.60–2.28]) and between AMD patients and PCV patients (*p* = 0.162; OR [95%CI] = 0.65 [0.36–1.19]). The frequency of TGTT deletion (AMD = 0.064. PCV = 0.655 & controls = 0.162) showed there was little difference among them. Therefore, the TGTT deletion allele had low association with AMD or PCV diseases.

### Linkage Disequilibrium (LD) and Haplotype Association Analysis

**Figure 1:**
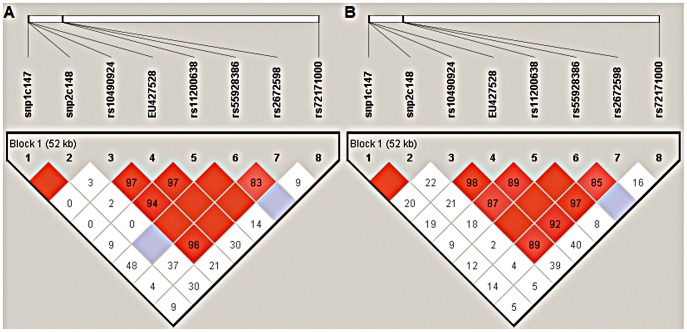
Analysis of pair-wise LD across *ARMS2* and *HTRA1* SNPs in northern Chinese PCV and nAMD cohort. A) Analysis of pair-wise LD across the eight *ARMS2* and *HTRA1* SNPs in northern Chinese PCV cohort. B) Analysis of pair-wise LD across the eight *ARMS2* and *HTRA1* SNPs in northern Chinese nAMD cohort. The relative physical position of each SNP is given in the upper diagram. The pairwise D′ between all SNPs is given below each SNP combination. And when D′ = 1.0, no number is given inside the square. Bright red squares indicate D′≥0.90 and LOD≥2. Bright red squares indicate D′<0.90 and LOD≥2. White squares indicate D′<0.90 and LOD<2.

Haplotype analysis revealed an extensive LD across all the 8 common polymorphisms, except rs72171000, in AMD (Figure 1A) and in PCV (Figure 1B).

Within the ARMS2 gene, pair-wise LD analysis in AMD cases showed rs10490924 was in high LD with EU427528 (310–311) in AMD (D′ = 0.97, 95% CI: 4.445–11.001; Fig. 1A) and in PCV(D′ = 0.97, 95% CI: 3.674–9.213; Fig. 1B). One significant association was noted for the TTG haplotype (P = 2.08×10^−18^) that was present approximately three times higher frequency (69.7% vs. 24.7%) in controls than in AMD and (65.7% vs. 24.7%) in controls than in PCV, indicating that it was risky.

Within HTRA1, among haplotypes defined by a risk SNPS(rs 11200638, rs 55928386, rs 2672598), a risk haplotype ACC and a non-risk haplotype GCC were significantly associated with both nAMD (*p* 1.42×10^−7^; OR 3.129, 95% CI: 2.033–4.816 and *p* 9.0×10^−4^; OR 0.422, 95% CI: 0.248–0.719, respectively) and PCV (*p* 1.0×10^−4^, OR 2.277, 95% CI: 1.498–3.461 and *p* 0.0019, OR 0.436, 95% CI: 0.258–0.737, respectively; Table 6).

**Table 6:** Haplotype analysis of *ARMS2* and *HTRA1* polymorphisms in exudative AMD and PCV.

No.	SNPs	Haplotype	Frequency			AMD-Control		PCV-Control		AMD-PCV	
	included		AMD	PCV	Control	p	OR(95%CI)	p	OR(95%CI)	p	OR(95%CI)
1.	rs10490924	G1	0.287	0.337	0.742	8.89×10^−19^	0.140	1.81 ×10^−14^	0.177	0.3093	0.792
	EU 427528						(0.089–2.20)		(0.112–0.280)		(0.504–1.243)
	(310–311)	T2	0.697	0.657	0.247	2.08×10^−18^	6.993	8.24×10^−15^	5.818	0.4194	1.202
							(4.445–11.001)		(3.674–9.213)		(0.769–1.878)
2.	rs 10490924	TA	0.697	0.614	0.244	1.92×10^−18^	6.953	2.37×10^−12^	4.691	0.1038	1.452
							(4.436–10.904)		(3.015–7.299)		(0.946–2.227)
	rs 11200638	GG	0.269	0.298	0.544	2.47×10^−8^	0.300	1.57×10^−6^	0.361	0.4941	0.871
							(0.195–0.460)		(0.236–0.552)		(0.556–1.346)
3.	rs 11200638	ACC	0.720	0.651	0.452	1.42×10^−7^	3.129	1.0×10^−4^	2.277	0.1557	1.374
							(2.033–4.816)		(1.498–3.461)		(0.884–2.136)
	rs 55928386	GCC	0.134	0.142	0.269	9.0×10^−4^	0.422	0.0019	0.436	0.842	0.944
	rs 2672598						(0.248–0.719)		(0.258–0.737)		(0.522–1.704)

* For the polymorphisms that were not a single nucleotide change, the wild type was denoted as 1 and the variant denoted as 2.

Moreover, haplotype analysis of rs10490924 and rs11200638 revealed that two haplotypes TA and GG were significantly associated with both nAMD (*p* 1.92×10^−18^, OR 6.953, 95% CI: 4.436–10.904 and *p* 2.47×10^−8^, OR 0.300, 95% CI: 0.195–0.460, respectively) and PCV (*p* 2.37×10^−12^, OR 4.691, 95% CI: 3.015–7.299 and *p* 1.57×10^−6^, OR 0.361, 95% CI: 0.236–0.552, respectively). There was no significant differences in haplotype frequencies between nAMD and PCV were also observed (*p* 0.1038, OR 1.452, 95% CI: 0.946–2.227 and *p* 0.4941, OR 0.871, 95% CI: 0.556–1.346, respectively).

We included the 8 polymorphisms used for haplotype analysis in logistic regression analysis. SNPs *ARMS2* rs10490924 and *HTRA1* rs11200638 were chosen for comparison since they showed strongest associations. Rs10490924 remained statistically significant in AMD (*p* 5.926×10^−5^) and in PCV (*p* 1.259×10^−5^) after adjusting for other SNPs, including rs11200638 (Table 7).

**Table 7:** Logistic regression analysis of SNPs in ARMS2 and HTRA1 between AMD and PCV.

	AMD				PCV			
	rs10490924		rs11200638		rs10490924		rs11200638	
	adjusted P	OR (95%CI)	adjusted P	OR (95%CI)	adjusted P	OR (95%CI)	adjusted P	OR (95%CI)
c.147G>T	4.980×10^−11^	13.937	0.002	3.354	2.488×10^−8^	7.912	3.755×10^−4^	5.096
		(6.352, 30.577)		(1.553, 7.244)		(3.823, 16.373)		(2.077, 12.501)
c.148 T>A	2.792×10^−10^	14.712	0.001	3.910	5.568×10^−7^	8.883	0.004	4.219
		(6.382, 33.912)		(1.724, 8.869)		(3.777, 20.891)		(1.571, 11.327)
rs 10490924	–		0.043	0.114	–	–	0.793	1.167
				(0.014, 0.931)				(0.367, 3.714)
EU427528	–		0.043	0.113	–	–	0.751	1.207
(310–311)				(0.014, 0.931)				(0.379, 3.845)
rs 11200638	5.926×10^−5^	63.749	–	–	1.259×10^−5^	7.752	–	–
		(8.390,484.385)				(3.092, 19.436)		
rs 55928386	5.097×10^−10^	12.602	0.005	3.192	1.190×10^−8^	9.241	0.001	4.624
		(5.668, 28.015)		(1.431, 7.119)		(4.302, 19.848)		(1.932, 4.066)
rs 2672598	2.735×10^−10^	17.192	0.001	4.298	6.003×10^−9^	10.400	1.715×10^−4^	6.961
		(7.109, 41.576)		(1.822, 10.141)		(4.724, 22.894)		(2.530, 19.152)
rs 72171000	5.361×10^−10^	23.736	4.144×10^−4^	4.301	6.846×10^−9^	9.549	2.597×10^−4^	4.622
		(8.733, 64.512)		(1.914, 9.666)		(4.451, 20.483)		(2.033, 10.510)

P value and OR (95%CI) of rs10490924 and rs11200638 after adjusting for the following SNPs.

### SNP rs10490924 does not Affect mRNA, Protein, or Surface Expression of ARMS2

**Figure 2:**
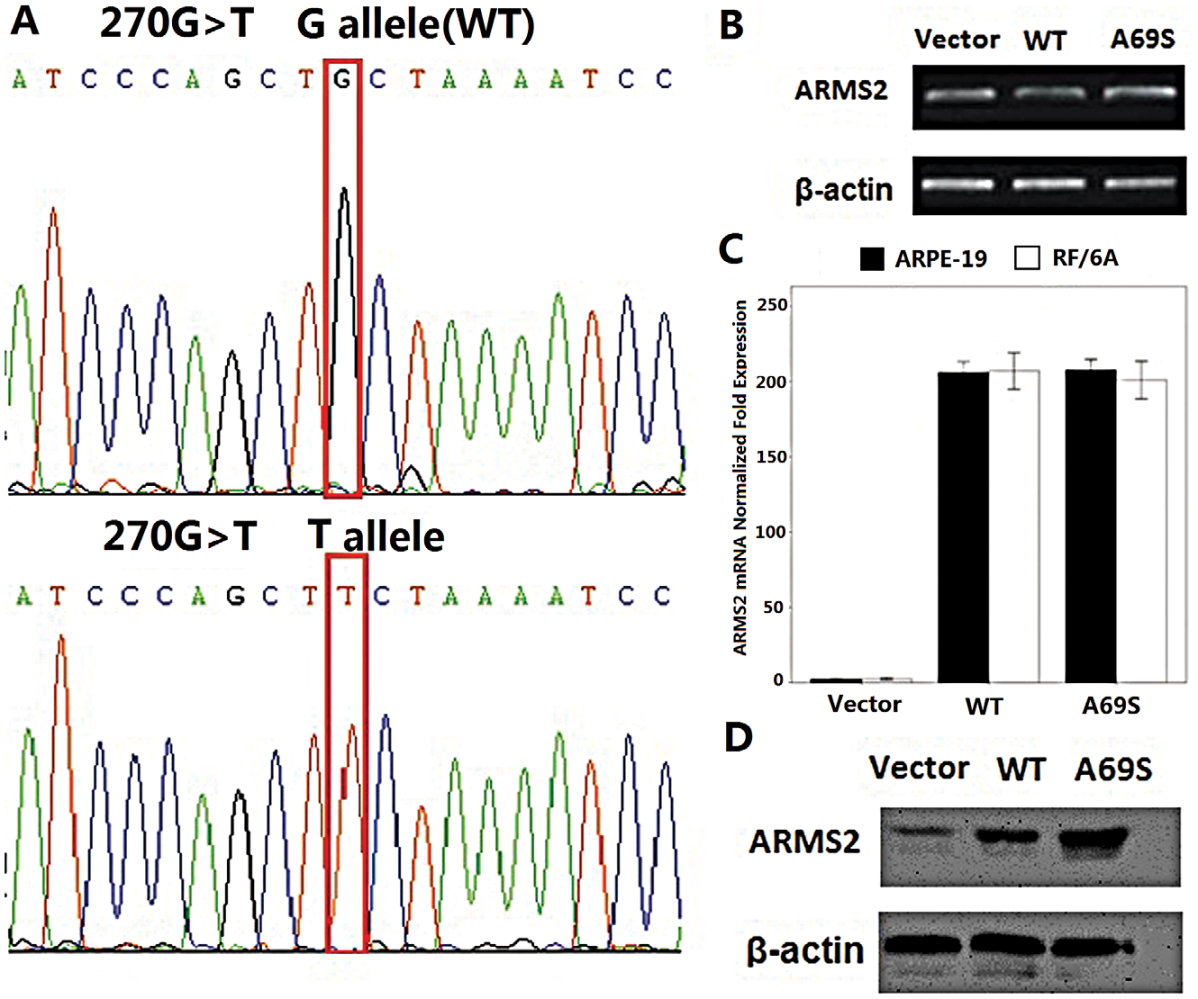
Rs10490924 (A69S in a coding change) does not affect mRNA, protein, or surface expression of ARMS2. A)Direct sequencing verified the wild-type (WT) and G270T ARMS2 plasmids. *B*) RT-PCR showed no significant difference between wild-type and G270T ARMS2 mRNA expression. *C*) Real-time RT-PCR confirmed the mRNA level finding. *D*) Western blot showed that the levels and migrations of the SNP mutant proteins were comparable with wild type. All experiments were repeated >3 times.

We next investigated the effect of the rs10490924 on ARMS2 expression. Wild-type ARMS2 and ARMS2 A69S mutation plasmids were verified before use by direct sequencing (Fig. 2*A*). Real-time PCR were performed to determine whether rs10490924 affected ARMS2 mRNA expression. No difference was found between ARMS2 wild-type and rs10490924 (Fig. 2*B, C*). To examine whether the rs10490924 affected protein expression, a Western blot was performed. β-Actin served as an internal loading control. The levels and migration of the mutant proteins were comparable with wild type (Fig. 2*D*).

### Effects of Wild-type ARMS2 and rs10490924 on the Proliferation of RF/6A Cells and ARPE-19 Cells

**Figure 3:**
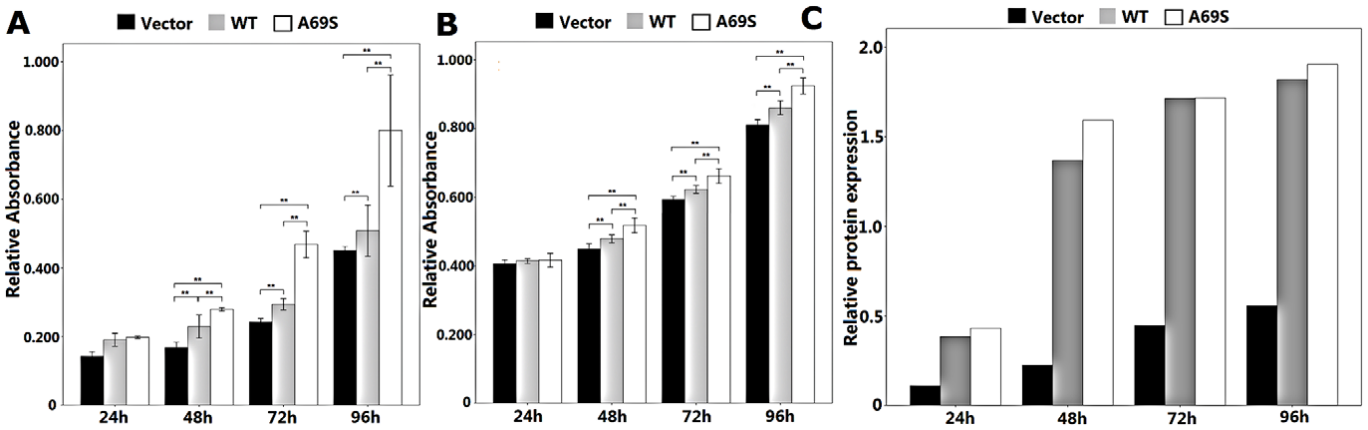
Effect of wild-type ARMS2 and rs10490924 on the proliferation of RF/6A and human RPE cells. RF/6A (**A**) and ARPE-19 (**B**) cell proliferation was measured with an MTT assay at 24 h, 48 h, 72 h, 96 h. Values are the means±SD of at least three independent experiments. Asterisks denote values significantly different from those of cells treated with wild-type ARMS2 and rs10490924 compared to negative control (p<0.01). (**C**)The time course of the ARMS2 protein expression profile mirrored that for ARMS2 protein expression levels after transfection in ARPE-19 cells. Abbreviations: wild-type ARMS2 plasmid-treated cells (WT); rs10490924 plasmid -treated cells (G270T); pReceiver-M29-Basic plasmid-treated cells (Vector) (*P<0.05, **P<0.01).

The up-regulation of wild-type ARMS2 and rs10490924 both promoted cell proliferation in RF/6A cells and ARPE-19 cells at 48 compared with the proliferation of the pReceiver-M29-Basic vectors as a negative control(p<0.01), and the up-regulation peaked on the fourth day (p<0.01). Compare with the wild-type, rs10490924 promoted cell proliferation more significantly (p<0.01; Figure 3A&B). The time course of the ARMS2 protein expression profile (Fig. 3C) mirrored that for ARMS2 protein expression levels after transfection in ARPE-19 cells.

### Effects of Wild-type ARMS2 and rs10490924 on the Attachment and Migration of RF/6A Cells and ARPE-19 Cells

**Figure 4:**
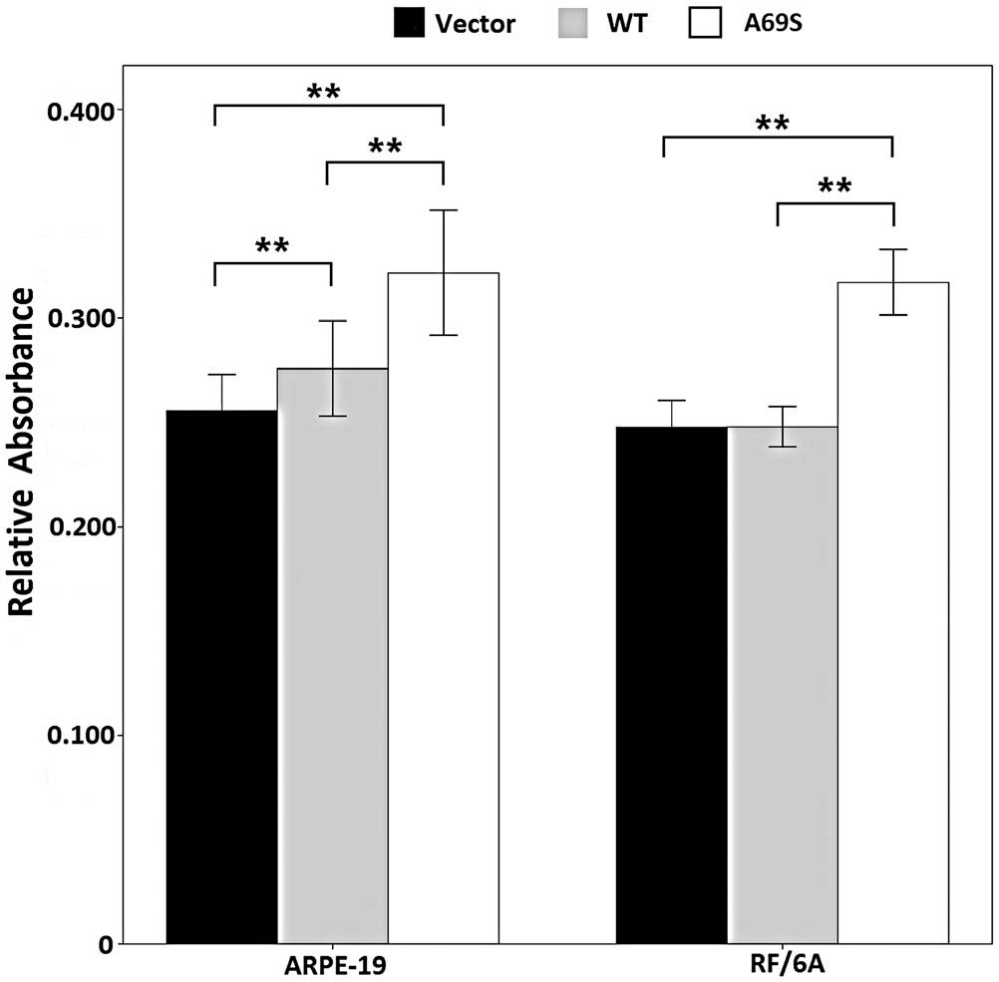
Effects of wild-type ARMS2 and rs10490924 on the attachment of RF/6A and RPE cells. Cell attachment was assessed after 6 h incubation and subsequent MTT assay. Values are the means±SD of at least three independent experiments. Asterisks denote values significantly different from those of cells treated with wild-type ARMS2 and rs10490924 compared to negative control (p<0.01). Abbreviations: wild-type ARMS2 plasmid-treated cells (WT); rs10490924 plasmid -treated cells (G270T); pReceiver-M29-Basic plasmid-treated cells (Vector) (*P<0.05, **P<0.01).

In the cell attachment assay, the rs10490924 increased the attachment capacity of RF/6A cells and of ARPE-19 cells (p<0.01; Figure 4) after 6h compared with that of the negative control. The negative control and wild-type ARMS2 groups were not significantly different in their capacities for cell attachment (p>0.05).

**Figure 5:**
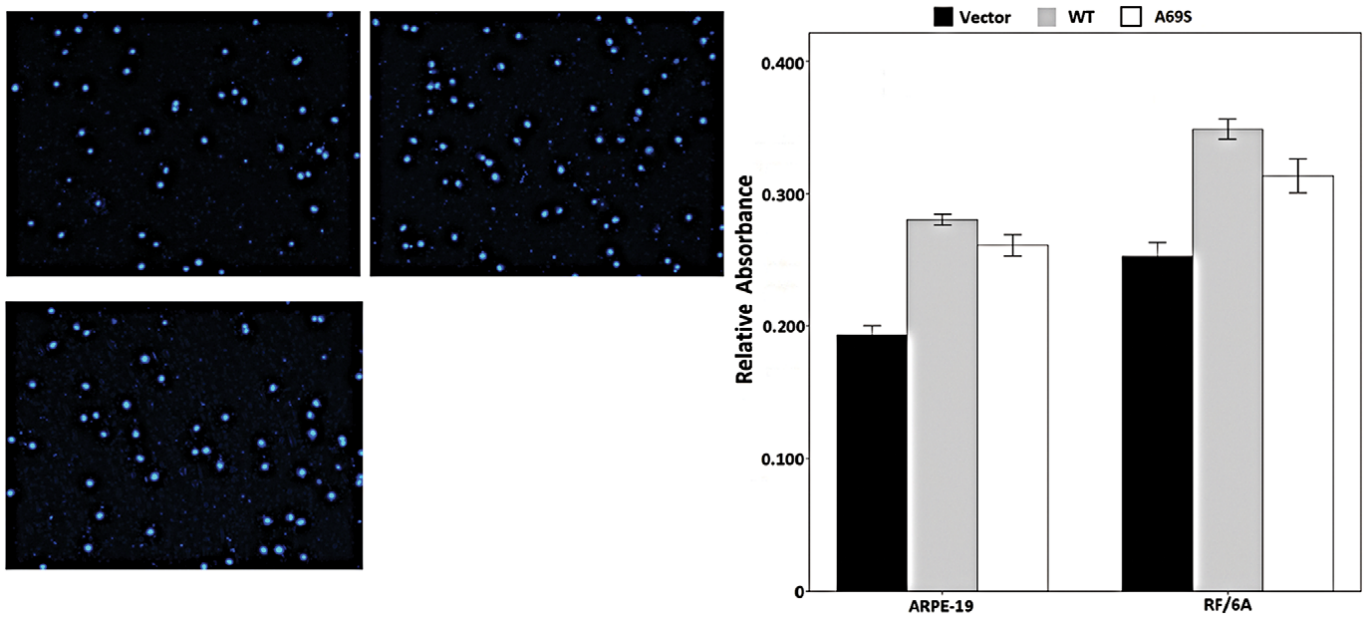
Effect of wild-type ARMS2 and rs10490924 on the migration of RF/6A and human RPE cells. The migratory activity of both cell lines was estimated based on the number of cells that had migrated through the filter of the chamber. A) Migrated cells of pReceiver-M29-Basic plasmid-treated RF/6A cells. B) Migrated cells of wild-type ARMS2 plasmid-treated RF/6A cells. C) Migrated cells of rs10490924 plasmid -treated RF/6A cells. Values are the means±SD of at least three independent experiments. D) The results showed that the number of migrating cells in the wild-type ARMS2 plasmid -treated group was the most during the three groups(p<0.01). Abbreviations: wild-type ARMS2 plasmid-treated cells (WT); rs10490924 plasmid -treated cells (G270T); pReceiver-M29-Basic plasmid-treated cells (Vector) (*P<0.05, **P<0.01).

Next, we explored the role of wild-type ARMS2 and rs10490924 in the migration of RF/6A and RPE cells using a modified Boyden chamber in which the RF/6A and ARPE-19 cells migrated through a porous membrane. As shown in Figure 5, the mean numbers of migrated cells among the wild-type ARMS2 and rs10490924-treated RF/6A and ARPE-19 cells were significantly higher than the number of migrated control cells (p<0.05), and then the mean numbers of migrated cells in the wild-type ARMS2 groups were highest during the three groups (p<0.05).

### Effects of Wild-type ARMS2 and rs10490924 on the Tube Formation of RF/6A Cells

**Figure 6:**
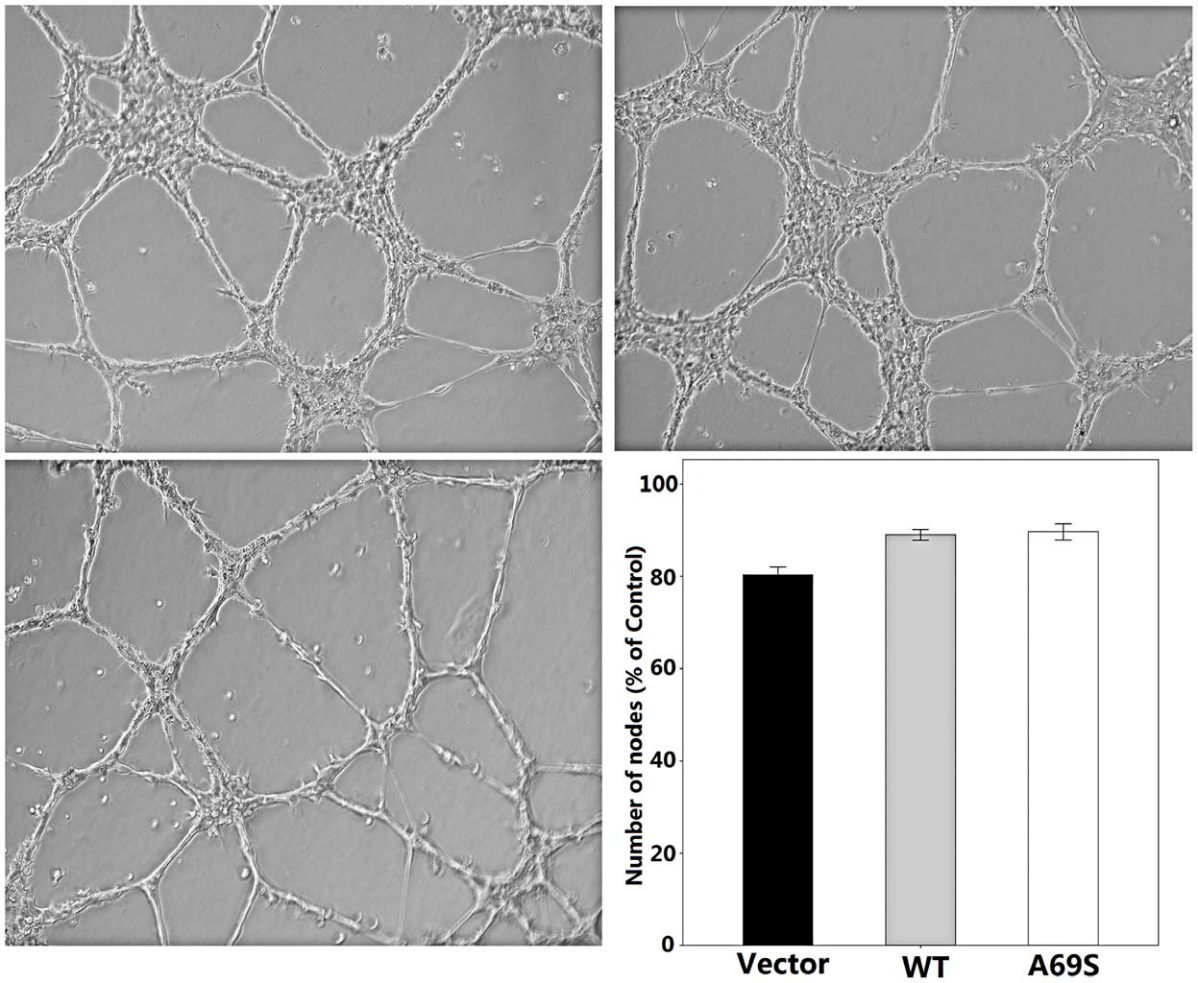
Effect of wild-type ARMS2 and rs10490924 on the tube formation of RF/6A cells. PReceiver-M29-Basic plasmid-treated RF/6A cells (A), wild-type ARMS2 plasmid-treated cells RF/6A cells (B) and rs10490924 plasmid -treated RF/6A cells (C) were plated on Matrigel as described in Methods. After 24 h of incubation, the three groups cells formed well organized capillary-like structures. Values are the means±SD of at least three independent experiments. There are no significant differenence during the three groups (D, p>0.05). Abbreviations: wild-type ARMS2 plasmid-treated cells (WT); rs10490924 plasmid -treated cells (G270T); pReceiver-M29-Basic plasmid-treated cells (Vector).

In a Matrigel assay, wild-type ARMS2 and rs10490924-treated RF/6A cells showed the same capacity to form a regular network compare with negative control. There was no significant difference during the three groups(p>0.05; Figure 6).

### Effects of Wild-type ARMS2 and rs10490924 on ARPE-19 Apoptosis and Cell Cycle

**Figure 7:**
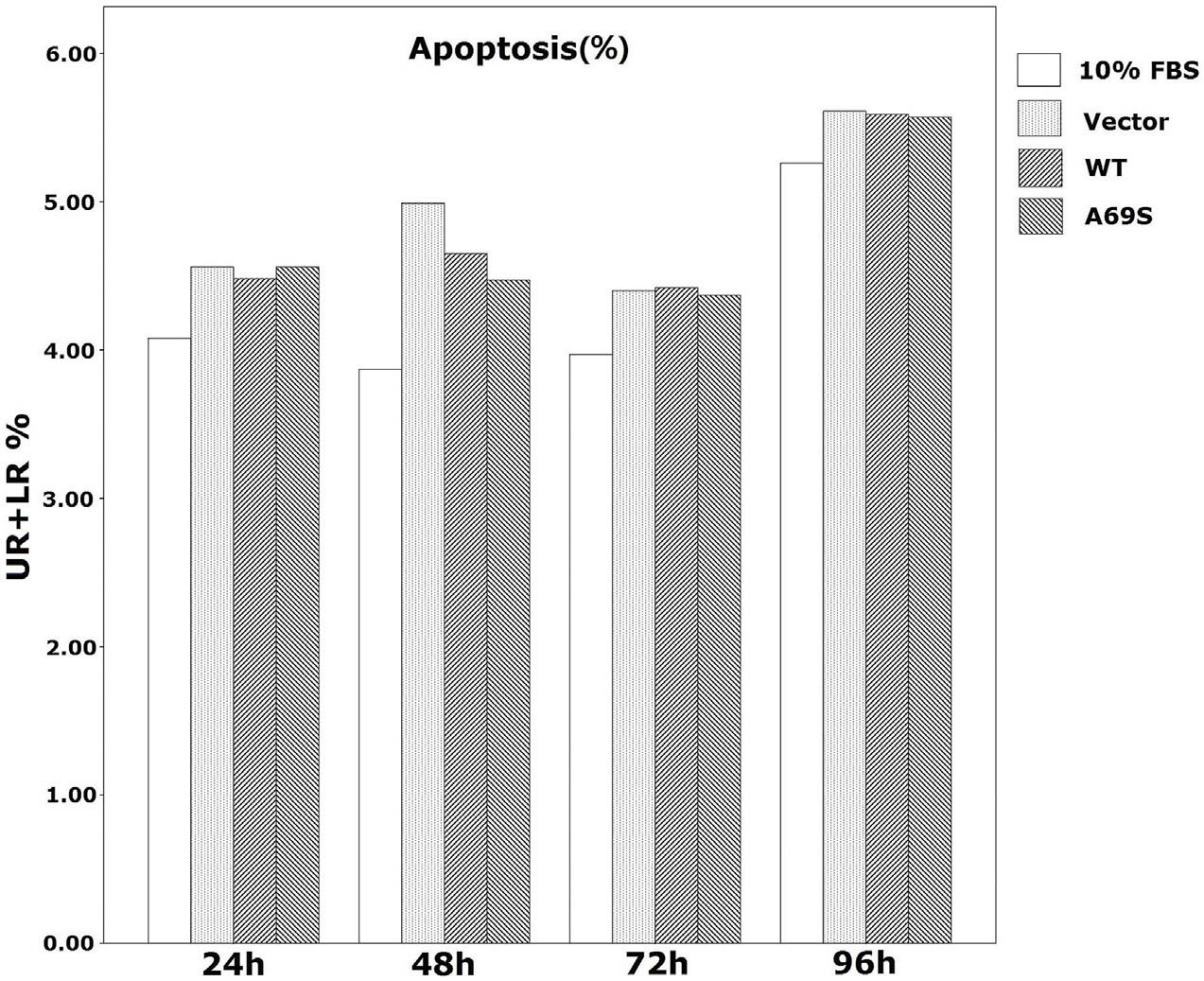
Effect of wild-type ARMS2 and rs10490924 on the apoptosis of human RPE cells. Apoptosis was quantified by flow cytometry measured by Annexin V and PI staining. Data are presented as mean±SEM.Each experiment was repeated at least three independent times. DMEM+10%FBS control was set to 100%.*P<0.05. UR: late apoptotic cells; LR: early apoptotic cells, UR+LR: apoptotic cells. Abbreviations: wild-type ARMS2 plasmid-treated cells (WT); rs10490924 plasmid -treated cells (G270T); pReceiver-M29-Basic plasmid-treated cells (Vector).

FACS was used to evaluate early and late apoptosis effects. As shown in Table 8 and Figure 7, after transfection for 24, 48, 72 h and 96 h, the early and late apoptotic ARPE-19 cells showed no significant differences during the three groups, with the percentage of apoptotic cells (UR+LR%) (p>0.05).

**Table 8:** Summary of flow cytomery data of apoSptosis measured by Annexin V and PI.

Time point	%	10%FBS	Vector	WT	A69S
24 h	UL	0.93±0.13	2.18±0.15	2.27±0.07	2.13±0.18
	UR	2.07±0.03	2.47±0.11	2.15±0.03	2.33±0.21
	LL	94.99±0.28	93.26±0.36	93.25±0.38	93.31±0.20
	LR	2.01±0.21	2.09±0.43	2.33±0.36	2.23±0.27
	UR+LR	4.08±0.20	4.56±0.20	4.48±0.35	4.56±0.19
48 h	UL	1.43±0.13	1.85±0.03	2.31±0.08	2.16±0.19
	UR	1.15±0.02	1.24±0.08	1.27±0.07	1.29±0.13
	LL	94.6±0.13	93.16±0.32	93.04±0.69	93.37±0.38
	LR	2.82±0.16	3.75±0.38	3.38±0.61	3.18±0.38
	UR+LR	3.87±0.14	4.99±0.32	4.65±0.61	4.47±0.52
72 h	UL	1.62±0.19	2.15±0.13	2.31±0.11	2.36±0.21
	UR	1.25±0.02	1.23±0.09	1.34±0.10	1.39±0.15
	LL	94.41±0.13	93.45±0.25	93.27±0.63	93.37±0.40
	LR	2.72±0.16	3.17±0.31	3.08±0.54	2.88±0.36
	UR+LR	3.97±0.16	4.40±0.32	4.42±0.53	4.37±0.43
96 h	UL	2.10±0.28	1.90±0.08	1.57±0.09	2.27±0.14
	UR	1.96±0.05	1.97±0.06	1.84±0.13	2.25±0.11
	LL	92.64±0.41	92.49±0.23	92.84±0.40	92.16±0.33
	LR	3.30±0.13	3.64±0.28	3.75±0.38	3.32±0.21
	UR+LR	5.26±0.15	5.61±0.27	5.59±0.48	5.57±0.25

**Figure 8:**
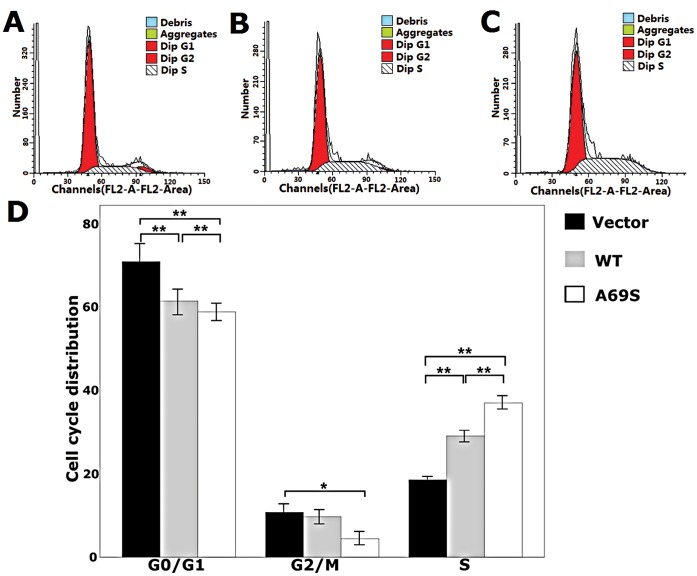
Effects of wild-type ARMS2 and rs10490924 on the cell cycles of human RPE cells. A) Cell cycle of pReceiver-M29-Basic plasmid-treated ARPE-19 cells. B) Cell cycle of wild-type ARMS2 plasmid-treated ARPE-19 cells. C) Cell cycle of rs10490924 plasmid-treated ARPE-19 cells. D) Data from the ARPE-19 cell cycle distribution of the control group, wild-type ARMS2 and rs10490924 group. Flow cytometric analysis demonstrates the effects of wild-type ARMS2 and rs10490924 on the human RPE cell cycle. The x-axis represents fluorescence intensity on a logarithmic scale and the y-axis represents the number of events. The results show that the fraction of cells in the G1 phase has decreased and the proportion of cells in the S phase has increased in the presence of wild-type ARMS2 and rs10490924-treated cells(*P<0.05, **P<0.01). Values are the mean±SD from three independent experiments. Abbreviations: wild-type ARMS2 plasmid-treated cells (WT); rs10490924 plasmid -treated cells (G270T); pReceiver-M29-Basic plasmid-treated cells (Vector).

As shown in Figure 8, the wild-type ARMS2 and rs10490924 resulted in a significant reduction of ARPE-19 cells in the G0/G1 phase and promoted an accumulation of cells in the S phase compared to controls in 48 h. Of the cells treated, 60.20% and 57.6% were in the G1 phase when treated with the wild-type ARMS2 and rs10490924, respectively, compared to 69.62% of the control group in the G1 phase (p<0.01; Figure 8). Moreover, 29.12% and 38.01% were in the S phase of the cell cycle, respectively, compared to 19.62% of cells in the control group (p<0.01; Figure 8). 10.76% were in the G2/M phase treated with the rs10490924 in the G2/M phase, respectively, compared to 4.39% of the control group in the G2/M phase (p<0.05; Figure 8) and there was no significant difference between the wild-type ARMS2 and control groups in the number of cells in the G2/M phase (p>0.05; Figure 8).

## Discussion

Neovascular AMD and PCV are important macular disorders sharing similar phenotypes and serious clinical complications, including hemorrhagic RPE detachment and vitreous hemorrhage [50], both of which have been used to classify PCV as a subtype of neovascular AMD [28]. However, there are discernable differences in their natural courses [51], responses to treatments and overall visual prognosis [52], indicating that PCV could be a type of macular disease that is different from AMD. Based on the reported AMD-associated genes [29], [33], [34], [39], genetic studies have been initiated to investigate the molecular mechanisms underlying the two diseases. Results of genotype analysis, variants at 10q26,have indicated that neovascular AMD and PCV may share common genetic background [38], [42], [53]. The associated variants at 10q26 overlap two known genes, *PLEKHA1*, *HTRA1*, and a predicted gene *ARMS2*. Each of these can have a plausible biological relationship to macular degeneration [28].

To clarify the genetic association and evaluate possible mechanism of disease susceptibility, we investigated the genetic profiles of nAMD and PCV through analysis of the *ARMS2/HTRA1* locus. A total of 8 polymorphisms in *ARMS2* and *HTRA1* were found to be associated with both diseases (Tables 2, 3, 4 and 5). Their genotype frequencies were all significantly different between nAMD and PCV (*p*<0.05). These results indicate resembling genetic effects in the *ARMS2/HTRA1* locus between the two diseases, but the size of the effects were different. Therefore, other genetic variations might also determine the development of exudative AMD and PCV. It is noted that, while the *p* values and ORs between the individual SNPs with AMD and with PCV may differ, the trend of associations remained the same (Tables 2, 3, 4 and 5). Therefore, the results showed that nAMD and PCV are subject to the same genetic influence as far as *ARMS2* and *HTRA1* SNPs are concerned.

In the study reported herein, the significant association was found in rs10490924 and EU427528 (310–311) in the ARMS2. For rs10490924, Our findings suggest risk allele is strongly associated with neovascular AMD and PCV, and with a stronger association in neovascular AMD than in PCV for northern Chinese population. This has been found in Janpanese and Caucasian [39], [41], [42], [54], [55]. Meanwhile, the almost same results were presented in EU427528 (310–311). a intron in ARMS2. Some reaseaches have mentioned that this risk allele of TG insertion had a strongly increased risk of developing AMD and PCV [43], [56]. Within HTRA1, there was statistical significance in rs55928386 and rs2672598 between AMD patients and controls, but not between PCV patients and controls. Therefore, the two allele had association with AMD, but not with PCV. Our findings also suggest that HTRA1 is involved in nAMD and in PCV for northern Chinese population. In the previous studies for the Japanese population, Kondo and Gotoh found that HTRA1 rs11200638 was significantly associated with PCV and nAMD although the odds ratios were higher for the nAMD cases than the PCV cases in Japanese [32], [38]. Lee comparing PCV and controls in Chinese population in Singapore showed rs11200638 to be significantly associated with PCV [31] and Liang found ARMS2 in southern Chinese remained significantly associated with AMD but not with PCV [57]. However, in our study we found the risk allele was tightly associated with AMD and PCV in northern Chinese population. The different results may be due to the different genetic background between southern and northern Chinese population in mainland China.

Our data indicate that both rs11200638 and rs10490924 share the same LD block which contains ARMS2 and HTRA1. This result is consistent with previous Caucasian studies [34], [39], [54]. There is high linkage disequilibrium (LD) across the *ARMS2*/*HTRA1* region, adding to the difficulty in identifying true causal variant(s) by association mapping alone [36]. The association signal at 10q26 converges on a region of an extensive LD block spanning *ARMS2* and *HTRA1* [36], [37]. This LD block harbors multiple susceptibility alleles of which the *ARMS2* rs10490924 has been reported to show the strongest evidence for association [37]. Two variants within this LD block that were correlated with A69S through strong LD–SNP rs11200638 in the promoter of HTRA1 [33], [34] and the insertion/deletion polymorphism (c.(*)372_815del443ins54) in the 3′-UTR region of *ARMS2* [*36*] –have recently been proposed as causal variants based on mechanistic functional evidence, but there is no agreement across studies [33], [34], [36], [37], [56], [58], [59]. Thus, the molecular basis of the susceptibility remains obscure.

To clarify the plausible biological function of wild-type ARMS2 and ARMS2 A69S mutation in AMD and PCV, we overexpressed these two genes in RF/6A cells and RPE cells as in vitro study model. Our findings showed that compare with wild-type ARMS2, A69S mutation resulted in a significant increase in proliferation and attachment but inhibited cell migration. However, neither wild-type ARMS2 nor A69S mutation affected cell apoptosis. Moreover, we found that neither wild-type ARMS2 nor A69S mutation affected tube formation of RF/6A cells. Tube formation is one of the main characteristics of retinal and choroid vascular endothelial cells, however, A69S mutation overexpressed RF/6A cells showed no significant difference with wild-type ARMS2 overexpressed ones on tubule formation. Therefore, neither wild-type ARMS2 nor A69S mutation might play a role in maintaining the tube-forming properties of RF/6A cells. Therefore, we thought neither wild-type ARMS2 nor A69S mutation had direct association with neovascularisation in the pathogenesis of AMD, which can explained ARMS2 has a significant association with pure dry AMD [60], [61]. Although we cannot formally reject the hypothesis that loss of LOC387715 is irrelevant to the disease, the spatiotemporal expression pattern of this gene and its exclusive emergence with the evolution of the macula in non-human primates, provide partial evidence for its role in AMD pathogenesis. We must note that AMD is a multifactorial disease with numerous susceptibility loci, therefore, the altered ARMS2 expression or function alone will not be sufficient to cause AMD.
